# Mandatory continuing professional development requirements: what does this mean for Australian nurses

**DOI:** 10.1186/1472-6955-12-9

**Published:** 2013-03-27

**Authors:** Kay Ross, Jennieffer Barr, John Stevens

**Affiliations:** 1 Gold Coast Campus, School of Health and Human Sciences, Southern Cross University, Locked Mail Bag 4, Coolangatta, QLD 4225, AUSTRALIA; 2 Lismore Campus, Health and Humans Sciences, Southern Cross University, Po Box 157, Lismore, NSW 2480, AUSTRALIA

**Keywords:** Mandatory continuing professional development, Nursing registration, Australia, Education, Clinical practice

## Abstract

**Background:**

This paper presents a discussion related to the recent decision in Australia to introduce mandatory Continuing Professional Development (CPD) for nurses. Historically there has been international debate surrounding mandatory CPD requirements; this debate is ongoing as Australian nurses face a diverse range of CPD offerings from a variety of providers.

**Discussion:**

The purpose of this paper is to examine how mandatory CPD requirements for national nursing registration in Australia have evolved and to present an analysis of what this will mean for Australian nurses. What is yet to be determined is how to measure professional development and the effectiveness of professional development education. This is important to the international community with consensus in the literature that professional development is linked to ongoing education. Contradicting arguments are presented about whether this professional development should be mandatory.

**Summary:**

Presenting a contemporary discussion about the current and potential impact of mandatory CPD requirements for nurses, this discussion paper utilises the case of Australia’s current national policy and CPD operation to examine the choices that nurses make in order to fulfil their legislative requirements. Additional arguments are presented about the barriers nurses face in undertaking CPD. The quest for effective CPD is complex and should incorporate different situations for nurses and individual learning styles.

## Background

This paper contributes to the global discussion about mandatory Continuing Professional Development (CPD) for health care professionals, particularly nurses. Amongst its many functions, CPD aims to sustain competence and introduce new skills as required for contemporary practice needs. CPD also offers the opportunity for nurses to “… maintain, improve and broaden their knowledge, expertise … and develop the personal and professional qualities required throughout their personal lives” [1]. In many countries ongoing investment in sustaining current evidence based knowledge to ensure appropriate provision of quality contemporary health care is considered a fundamental ethical obligation for all nurses [2-7].

Human resources are seen as the most important contribution to health care [8]. As knowledge changes and new tools, technologies and procedures are developed, on-going education and training for health care professionals is seen as a key investment strategy [9]. A great deal of literature [2,4,10-14] has focused on appropriate educational approaches to ensuring effective CPD.

Moves towards national registration and CPD requirements for nurses and other health professionals have been considered for many years in a number of countries, including Australia. This has led to the decision from the Coalition of Australian Governments (COAG) [15] stating that “COAG agreed to the introduction of a national registration and accreditation system for health professionals”. The Nursing and Midwifery Board of Australia (NMBA) responded to this new legislation and as a result of this decision, from July 2010 Australian nurses are required to complete a minimum 20 hours of CPD as part of their yearly registration renewal [16].

There has been much debate about the introduction of mandatory CPD requirements for nurses and other health professionals. The literature about professions, which spans many decades, has consistently noted that the need for commitment to continued professional development [17-20] is essential for ongoing learning. For nursing to be credited with the status, authority and autonomy that accompanies a profession, then mandatory professional development has always been inevitable [21].

Hamilton [22] found that mandatory CPD for nurses was not new and that the issue of professionalism needs to link to ongoing education and learning and should be an obligation for all nurses. Research by Gopee [23] emphasises the need for nurses to participate in lifelong learning and continuous professional education in order to keep their knowledge and skills up to date, rather than just being competent. Thomas [24] agrees, saying that nurses need ongoing education in order to provide consistently high level patient care.

Whilst CPD is not a new concept it is not well understood in some health professions, particularly nursing in Australia [25]. This may be due to the ad hoc process of undertaking CPD, with states and territories of Australia in the past having different legislation about continuing education. Historically some nursing specialty groups required CPD in order to be credentialed (e.g. Mental Health Nurses); however the majority of nurses were not obliged to undertake any specific CPD activities. CPD requirements were not formalised and there were no agreements of the content or extent that nurses and midwives needed to sustain knowledge of current evidence for their professional practice. Therefore, the present situation in Australia is that most health care professionals are now obliged by legislation to engage in mandatory continuing education in order to sustain practice registration. This is of interest to the international community, as mandatory CPD continues to be considered or rejected in other countries around the world [3,5,26-38].

## Methods

The search strategy that was used to inform the discussion included the data bases of CINAHL plus, Med-line, ERIC and Google Scholar databases. The following search terms were used: ‘nurs*’AND ‘CPD’, ‘professional development’, ‘inservice’, ‘education’ ’training’, ‘mandatory’, ‘workplace training’, ‘lifelong learning’, ‘motivation’, ‘barriers’. The search was supplemented using citation tracking and secondary references. The initial search resulted in 112 articles. The reference list of articles that appeared to meet the study criteria were examined to identify further literature and a further 17 were identified. Additionally a search of online theses, using Google Scholar, resulted in nine theses. A total of 29 articles and two theses that met the criteria were included in the study.

The sample consists of studies examining what motivates nurses to attend/complete CPD. Inclusion criteria were as follows: (1) published between 2002 and 2012 (2) located through a computerized search of CINAHL, Med-line, ERIC and Google Scholar (3) published in English (4) designed to explore the reasons why nurses participate in CPD (5) examined impact of mandatory CPD on nurses’ participation in CPD.

## Discussion

One purpose to undertake CPD is for the provision of effective health care. The current international context of clinical practice includes the need to respond quickly to change in order to maintain competence and provide appropriate health care. However, attending CPD does not alone guarantee sustaining competency.

The type of mandatory CPD, how it is implemented and evaluated to show the efficacy of this education and learning, and in turn how effectively this is applied in practice is a contentious debate [5,39,40]. Historically a great deal of discussion has occurred arguing whether nurses should be required to participate in mandatory CPD [22,24,41-50]. Currently there are no guidelines of what the CPD needs to consist of except that all health professionals tend to predominantly focus learning towards knowledge relevant to their current practice. Additionally, barriers to people engaging in CPD are also common. This discussion will first commence with defining CPD.

### CPD defined and described

The literature includes a number of terms used interchangeable with CPD. These include CPE (Continuing Professional Education) [42], In service, [51,52] CE (Continuing Education),[53,54] Lifelong Learning [55,56] and PD (Professional Development) [57,58]. Most of these definitions refer to professional education gained after professional entry education [22] and do not refer to post-graduate studies leading to advanced diplomas or degrees.

The recent debate about CPD specifically for Australian nurses has proposed that it needs to be relevant to the context of practice (NMBA 2010). This debate has recognised that clinical practice is greater than the act of providing health care but rather could also include areas that influence the provision of health care. For example, the Australian Nursing & Midwifery Competencies state that:

Nurses are personally accountable for the provision of safe and competent nursing care. *It is the responsibility of each nurse to maintain the competence necessary for current practice* (authors’ emphasis). Maintenance of competence includes participation in ongoing professional development to maintain and improve knowledge, skills and attitudes relevant to practice in a clinical, management, education or research setting [59].

Professional development opportunities need to engage health care professionals in a process of growth. Professionalism in contemporary society includes the ability to respond quickly to change. Such change can originate from government policy, new health service provision or from market conditions where health care needs to change to respond to new health issues [60]. In Australia, an example of responding to a new health issue has been the clinical presentation of the Hendra Virus, a recently identified new infection that has significant mortality and morbidity associated with human exposure.

Ever changing technology is a constant demand for adaptation by nurses. With increasing health care costs, the international health community responds with a variety of management strategies. However, any change or new situation provides an opportunity for new learning where things are seen from different perspectives. For example, recipients of CPD can become aware of and value their own contributions to the profession or respect work by colleagues both within that profession as well as other health care disciplines [12].

### Current state of CPD offered in Australia for nurses

There is a myriad of CPD opportunities for nurses in Australia, offered by a variety of companies, universities, Technical and Further Education (TAFE), special interest groups and individuals. A “Google” search (5 December 2012) for “CPD Australia nurses” revealed 557,000 results; while these results show some duplication of entries, it does indicate the extensive array of CPD offerings available for nurses in Australia (65% of people who look for a service or product use Google as the first step in identifying what is available and where they might find the information that they need [61]). It is estimated that the CPD industry for registered nurses alone to be worth $180m per year (from a needs analysis undertaken by the authors; RNs are reporting spending on average $30 for each CPD hour; these figures do not include any CPD that may be offered at no cost by the nurse’s employer e.g. mandatory skills acquisition such as manual handling, Occupational Health & Safety etc.). Currently, there are approximately 300,000 nurses in Australia [62], each requiring a minimum of 20 hours CPD per year. This equates to a potential multi-million dollar industry for providers of CPD to nurses in Australia.

Companies that are currently providing CPD opportunities for nurses include those focused primarily for the purpose of professional development (e.g. AUSMED), and those that offer CPD for nurses as an adjunct to their core business (The Australian College of Nursing), Staffing Agencies (e.g. CQ Nurse), Aged Care Providers (e.g. Frontline Care Solutions), Nursing Unions/Associations (e.g. Australian Nurses Federation, NSW Nurses Association), nursing specialty groups (e.g. The Australian College of Mental Health Nurses), TAFE , universities (e.g. Southern Cross University) and a variety of individuals and groups of nurses and other training/allied health professionals.

Currently there are no guidelines addressing what type of organisation can offer CPD training for nurses and who can deliver such education. The only accrediting body in Australia is the Royal College of Nursing (RCNA) (now known as The Australian College of Nursing) who offer “endorsement”. However, there is no requirement that CPD activities are endorsed or approved by any organisation or body. The Australian College of Nursing (ACN) website states that:

1. “Endorsement by RCNA is a quality assurance process; it is a guarantee that an

2. Educational activity has been scrutinised by RCNA and approved as meeting our

3. Professional standards and requirements for nurse education” [58].

However, not all providers are endorsed by ACN and there are no requirements to do so. Nurses are free to choose who provides their CPD requirements, the topic and content (as long as it is relevant to their practice) and the mode of delivery (face-face, online, distance).

Some of the education providers are also RTOs (Registered Training Organisations) which provide specific vocational education or assessment services, as well as CPD activities which nurses may choose to complete. There are a number of companies which provide RTO activities including TAFE institutes, private and community providers, schools, higher education institutions, industry organisations and enterprises [63].

As can be seen from Table 1 (below), there are many differences between training offered by an RTO and those endorsed by the ACN. An RTO offers nationally recognised qualifications in a variety of areas including nursing and health. These can range from a Certificate II in Disability Studies through to a Diploma of Nursing (Division 2). RTOs may also offer short courses which do not lead to a formal qualification (e.g. Certificate in Wound Care).

**Table 1:** **Comparison of ACN and RTO [**[58]**,**[63]**]**

	**ACN (RCNA)**	**RTO**
National recognition	National recognition by nursing bodies	National recognition of qualifications by the tertiary and health sector
Quality assurance	Quality assurance process approved as meeting professional standards and requirements for nurse education	Training is delivered by an organisation which meets national standards
Qualifications	Provides endorsement only	Qualifications are based on identified industry needs
Recognition of Prior Learning	Some courses may be recognised for recognition of prior learning (RPL) under separate agreements with the tertiary sector	Access to recognition of prior learning (RPL)
Articulation into higher education	Some providers also have a Memorandum of Understanding (MoU) with tertiary institutions	Opportunities for articulation into further training (this may include higher education)

The ACN endorses courses which are then able to state that they have been “Endorsed by the ACN”. Endorsement means that the course has met the criteria as set down by the ACN; however it does not mean that there is any formal qualification associated with it, and as stated previously, there is no requirement for nurses to choose an “endorsed” CPD activity (see Table 1).

Whilst this section only discusses specific Australian conditions for nurses, CPD is relevant to all countries as the CPD industry has potential financial rewards, whilst balancing the costs to develop and implement CPD activities [33,64]. Also relevant to all countries is the claim that effective CPD is dependent on availability and quality of the program that is provided. Of great importance, is that the consumer, the recipient of CPD, needs to understand the language associated with CPD, such as the term endorsement and how this relates to their professional registration.

### CPD delivery modes

#### Online learning

A growing trend in CPD offerings is online learning. Online learning (or “e-learning”, “web based training” etc.) includes webinars (participants log into a “live” online presentation), e-courses based on a Learning Management System (LMS) (e.g. “Blackboard”, “Moodle”), PowerPoint presentations, lectures, notes, pod casts, workshop recordings and quizzes.

Online learning offers a range of potential benefits including reduced additional cost (no accommodation, parking, transport or child minding costs), reduced time (eg. no travel time) and flexibility (complete at times suitable for participant). Online learning is a viable option for nurses working shift work, with family responsibilities and/or who work in rural areas. However, disadvantages may include the need for a computer with internet access (preferably Broadband) and internet skills, including internet browsing, uploading documents and online communication (forums and online discussion rooms). Additionally, online learning that can be accessed globally will need to be applicable and appropriate for specific country educational needs. For example, one educational program could lead to a qualification in one country but not in another.

The cost of these online courses varies considerably, ranging from $30.00 (AUS) for a one hour session and up to hundreds of dollars for longer or ongoing training. Some companies offer “membership” training starting at $150 (AUS)/yr. These membership sites offer a range of CPD Courses and participants can choose from a variety of offerings in order to meet their yearly CPD requirements. Some online providers offer discounts for “group” bookings (e.g. five or more participants registering together receive 10% discount).

Irving et al. [65] highlighted some of the issues faced by nurses wanting to update their skills and knowledge including many nurses finding it difficult to attend traditional face-face training because of staff shortages and geographical isolation They concluded that the profession needs to explore other options, including e-learning which offers a number of advantages including flexibility and accessibility [65].

#### Face-face learning

Face-face CPD opportunities include workshops, seminars, conferences, lectures and mandatory training which are offered onsite at the workplace or offsite (locally, nationally and internationally). Costs start at $60 (AUS) for a 3 hour workshop, $200 (AUS) for one full day seminar and $800 (AUS)/day for conferences, with costs going as high as thousands of dollars for National and International Conferences and Seminars [66-69]. Added costs include travel, accommodation, time off work and child minding, all of which can add hundreds-thousands of dollars to the total outlay.

### Challenges of CPD

One of the purposes of CPD is to maintain competent practice through developing knowledge and skills ([70]:142). Underlying the introduction of mandatory CPD is the assumption that it:

Is the means by which members of the profession maintain, improve and broaden their knowledge, expertise and competence, and develop the personal and professional qualities required throughout their professional lives. The CPD cycle involves reviewing practice, identifying learning needs, planning and participating in relevant learning activities, and reflecting on the value of those activities”[1,4,16].

The current mandatory CPD in Australia only states a number of hours and not the frequency of engagement. Yet research has shown that retention of clinical skills and knowledge are dependent on regular education and revision [70]. Additionally, what is not clearly evident from research findings is the conditions required for effective CPD. For example, what can be defined as “regular”, which will in turn lead to effective education? Additionally, attendance at CPD sessions does not guarantee that learning has taken place and when assessment is involved this may not lead to maintaining competence [13,71]. More importantly, at this point, there is little evidence that professional development will improve patient outcomes [19,72].

Therefore, the issue of the quality of CPD offerings is one that does need to be addressed. Some nurses focus on “getting their hours up” rather than addressing their learning outcomes and relevance to their area of practice. Despite the fact that quality control should be an essential aspect of any course-development process [65], there are currently no quality requirements for CPD offerings for nurses in Australia nor for many other countries.

### Barriers to CPD

Understanding the barriers to CPD has been a major discussion in the literature [60,73,74]. Research examining barriers have included national and state-wide research undertaken looking at barriers to nurses engaging in CPD [75,76]. Other research has examined the barriers for nurses to engage in CPD who reside in rural and remote localities [42,77,78]. The reasons noted for lack of engagement in CPD are consistent across the literature, regardless of country and geographical location.

In particular, financial constraints due to lack of funding and personal costs has been noted as a significant barrier [79-82]. Nurses are requesting financial support for reasonable costs associated with professional development activity [82]. However this often is not available and many nurses self-fund their own training.

This lack of support by employer occurs regardless of the acknowledgement of both employer and nurses for the need for professional development. Lack of financial support relates to the lack of time available during work due to work load, as well as staff not being replaced when away on CPD activities and this has been noted as a significant issue [79-81]. Computer and internet access at the workplace is seen as important for CPD activity and yet lack of such access is noted as a common barrier in the literature [79,82]. Additionally lack of technical support at work was seen as a significant barrier with nurses noting that equipment and software for CPD learning is constantly changing and becoming outdated [82].

Personal factors associated with barriers to CPD engagement has also been a consistent theme discussed in the literature referring to on-going professional development of nurses. Limited access to childcare is a key concern for parents but caring for other dependents is also a barrier to finding time for CPD [79,81]. Participant’s energy and motivation was noted as both being a benefit but also a barrier and was also linked to self-efficacy associated with CPD [79,81,82].

The lack of appropriate and accessible professional education is one of the key frustrations reported by nurses in the literature. Lack of transport or unavailability of nearby education providers for CPD, particularly those in rural and remote localities will inhibit engagement [54]. The need for appropriate professional development, particular for clinical relevant learning was an important issue for nurses [80,82]. Currently there is a deficit of CPD that includes material for different learning styles [80,82]. Kataoka-Yahiro and Mobley [82] also highlighted that appropriate CPD should include consideration of generational differences in learning styles.

## Summary

This paper presents a contemporary discussion about mandatory CPD using the case of Australia’s current national policy and CPD operation for nurses. Whilst Australia has taken the step to mandate an approach to nurses engaging in CPD, this paper highlights that there is still a great deal of research that should be undertaken to ensure effective education and achieve the goal of sustaining competency, knowledge and skills in practice. Issues, including the quality, content and evaluation of CPD are areas that need to be addressed. Mandatory CPD requirements for Australian nurses will not be enough to achieve CPD aims. The challenge now is to ensure that nurses have access to equitable, relevant and professional CPD opportunities.

## Abbreviations

ACN: Australian college of nursing; ANF: Australian nursing federation; ANMC: Australian nursing & midwifery council; AUS: Australian dollar; COAG: Coalition of Australian governments; CE: Continuing education; CPE: Continuing professional education; CPD: Continuing professional development; LMS: Learning management system; NMBA: Nursing & midwifery board of Australia; PD: Professional development; RCNA: Royal College of nursing Australia; RPL: Recognised prior learning; RTO: Registered training organisation; TAFE: Technical & further education.

## Competing interests

The authors declare that they have no competing interests.

## Authors’ contributions

KR carried out the literature review, developed the outline, wrote the draft and the final version. JB helped develop the manuscript, reviewed the draft and contributed to the final version. JS contributed to the discussion on the state of CDP in Australia and provided feedback on the draft and final version. All authors read and approved the final manuscript.

## Authors’ information

Kay Ross is a Lecturer in the School of Health and Human Sciences (Nursing) at Southern Cross University, QLD. She has worked in Continuing Professional Development at the College of Nursing (Sydney) and contributed to the CPD Program at Southern Cross University

Dr Jennieffer Barr is currently the Director of Higher Degrees Research Training for the School of Health and Human Sciences, at Southern Cross University, QLD

Dr John Stevens is the Director of Post Graduate and Corporate Health Education and Development in the School of Health and Human Sciences at Southern Cross University, NSW.

## Pre-publication history

The pre-publication history for this paper can be accessed here:

http://www.biomedcentral.com/1472-6955/12/9/prepub
